# Inhibition of mechanical stress-induced hypertrophic scar inflammation by emodin

**DOI:** 10.3892/mmr.2015.3265

**Published:** 2015-01-28

**Authors:** CHENG LIU

**Affiliations:** Department of Plastic Surgery, Jiangxi Provinicial People’s Hospital, Nanchang, Jiangxi 330006, P.R. China

**Keywords:** hypertrophic scarring, emodin, inflammation, phosphoinositide 3-kinase/Akt

## Abstract

At least 50% of hypertrophic scarring (HS) is characterized by inflammation, for which there is currently no effective treatment available. Emodin is a major component of the widely used Chinese herb, rhubarb, which has been used to treat inflammation in several types of disease. However, few studies have investigated the efficacy of emodin in the treatment of HS. In the present study, a mouse model with mechanical stress-induced HS was used to investigate the effects of emodin (20, 40, 80, or 120 mg/ml) on HS, and to determine the potential underlying mechanisms. Treatment with emodin significantly attenuated HS inflammation, as determined by histopathological assessment of the scar elevation index, collagen structure and inflammation (P<0.05). Furthermore, treatment with emodin (40 mg/ml) markedly inhibited phosphoinositide 3-kinase (PI3K)/Akt activity (P<0.01) and this attenuation was associated with reduced expression levels of tumor necrosis factor-α, interleukin-6 and monocyte chemoattractant protein-1 (P<0.05) in the HS tissue. The results of the present study indicated that administration of emodin had therapeutic effects on the progression of HS and the underlying mechanism of this may be due to inhibition of the PI3K/Akt signaling pathway.

## Introduction

Hypertrophic scarring (HS) results from fibroblast proliferation and activation and involves the deposition of a substantial quantity of abnormally secreted extracellular matrix (ECM) proteins and the secretion of several types of cytokines and chemicals. HS is regarded as a multifactorial fibrotic disorder; however, the specific mechanisms underlying fibrogenesis remain to be elucidated (1). Enhanced and prolonged inflammation is a common pathogenic feature of HS, which has been described in previous studies (2–4). Numerous therapeutic approaches, including surgical excision, hormone therapy, cytotoxic drugs, superficial compression therapy and radiation therapy, have been used in the clinical treatment of HS (5). However, none of these therapeutic approaches provide effective results.

Topical inflammation contributes markedly to the formation of HS. During the early proliferation stage, the infiltration and retention of inflammatory cells can be observed by histological examination (6). The primary inflammatory cells involved are monocytes, lymphocytes and mast cells (7). In addition, several types of inflammatory cytokine, including monocyte chemoattractant protein (MCP)-1, tumor necrosis factor (TNF)-α and interleukin (IL)-6, are secreted by these inflammatory cell and mediate the interaction of inflammation and fibroblast activation. These processes, combined with mechanical forces, are important elements, which lead to the formation of HS (8–11). Therefore, since the inflammatory response has an important role in the development of HS, treatment that is able to terminate the HS inflammatory response offers a potentially promising therapeutic approach in the treatment of HS.

Emodin is a major component of the widely used Chinese herb, rhubarb. It has been used to treat several diseases, including inflammation and cancer. The potential therapeutic effects of emodin have been investigated in pancreatitis, asthma, arthritis, atherosclerosis, myocarditis, glomerulonephritis and Alzheimer’s disease (12,13). The signal transduction pathways affected include nuclear factor-κB and phosphoinositide 3-kinase (PI3K)/AKT (13). Previous studies have demonstrated that emodin can markedly suppress *in vitro* fibrotic activities of rat kidney fibroblasts and hepatic stellate cells (14,15). However, few studies have examined the effectiveness of emodin in the treatment of HS.

Based on previous findings, the present study hypothesized that emodin may have a positive effect on HS by attenuating the HS inflammatory response. Therefore, the aim of the present study was to investigate whether emodin can be used as an effective drug for the treatment of HS.

## Materials and methods

### HS models and materials

Female wild-type C57BL/6 mice (eight-weeks-old) were purchased from the Shanghai Laboratory Animal Center (Shanghai, China). All the mice were maintained under standard conditions at 23–26°C, 12-h light/dark cycle, 150 lux with access to standard food and clean water *ad libitum*, according to the guidelines approved by the Shanghai Jiao Tong University Animal Care and Use Committee (Shanghai, China). The study was approved by the medical ethics committee office of Jiangxi Provinicial People’s Hospital, Nanchang, China.

Hypertrophic scar models were created, as described previously (16). Briefly, two 2 cm linear full-thickness incisions (1.25 cm apart) were made on the dorsal midline of each mouse and sutured using 4-0 silk threads (Shanghai Gold Medical Supplies Co., Ltd, Shanghai, China). Subsequently, 4 days post-incision, the sutures were removed from the scars and two mechanical stress devices, produced by our lab based on a previously published method (16), were sutured overlying the incisions. Equal stretching forces were applied between days 4 and 14 post-incision in one of the two scars of each mouse and the other scar remained untreated. A total of 20 mice were divided into two groups, one group was intraperitoneally injected with emodin (10 mg/kg; Sigma-Aldrich, St Louis, MO, USA) and the other group was intraperitoneally injected with an equal quantity of Dulbecco’s modified Eagle’s medium (DMEM; cat. no. 11965; Gibco-BRL, Carlsbad, CA, USA) once each day between days 4 and 14 post-incision. Half of the mice from each group were sacrificed at day 14 and the other half were sacrificed at day 28 post-incision. Mice were sacrificed by cervical dislocation. Scar specimens and normal specimens were then harvested for further analyses.

### Cell culture

Hypertrophic scarring fibroblasts (HSFs) and normal fibroblasts (NFs) were obtained from the above-described specimens using trypsin (Trypsin-EDTA, 0.05%; Gibco-BRL), and cultured in DMEM supplemented with 10% fetal bovine serum (Gibco-BRL), 100 U/ml penicillin and 100 mg/ml streptomycin (Beijing Solarbio Science & Technology Co., Ltd., Beijing, China). THP-1 human acute monocytic leukemia cells were obtained from the American Type Culture Collection (Manassas, VA, USA) and maintained in RPMI 1640 medium (Gibco-BRL) supplemented with 10% fetal bovine serum, 100 U/ml penicillin, 100 mg/ml streptomycin and 0.5 mM β-mercaptoethanol (Gibco-BRL). The HSF and NF cell lines were incubated at 37°C in a humidified atmosphere containing 5% CO_2_. Primary fibroblasts between passages six and eight were used.

### Total protein assay

Following treatment with 20, 40, 80 or 120 *μ*g/ml emodin for 24 h, identical numbers of HSFs (4×10^5^ cells) were harvested. The microplate bicinchoninic acid (BCA) method was used to determine the quantity of total protein using a BCA Protein Assay kit (Pierce Biotechnology, Inc., Rockford, IL, USA) according to the manufacturer’s instructions.

### Histopathological examination

The specimens from the HS murine models were incised, fixed in 10% neutral-buffered formaldehyde, embedded in paraffin and stained with either hematoxylin and eosin or picrosirius red (Fluka Analytical, Buchs, Switzerland). The scar elevation index (SEI), denoting the ratio of scar thickness to the thickness of the adjacent normal skin (17), the collagen structure and the level of inflammation of the HS tissue, was assessed via examination of the stained sections and scores between 0 and 3 were assigned for SEI: 0=SEI 0–1, 1=SEI 1–2, 2=SEI 2–3 or 3=SEI≥3; collagen structure: 0=well organized with no whorl; 1=disorganized with no whorl; 2=disorganized with one whorl/high power field (HP); or 3=disorganized with ≥two whorls/HP and inflammation: 0=no monocytes; 1=monocyte or mast cell 2–5/HP; 2=monocyte or mast cell 5–10/HP or 3=monocyte or mast cell >10/HP. The scores for each factor were then added together to calculate the histopathological scores of the specimens.

### Adhesion assay

Adhesion assays were performed, as described previously (17,18). The HSFs obtained from the DMEM treated mice were seeded at 3×10^5^ cells/well in 24-well plates and grown to 80% confluence. The THP-1 cells were fluorescently labeled using 2.5 mM calcein AM (Dojindo Molecular Technologies, Inc., Kumamoto, Japan). Subsequently, a 500 *μ*l THP-1 cell suspension at a density of 1×10^5^ cells/ml in 20, 40, 80 or 120 *μ*g/ml emodin, was added to each well. The plates were centrifuged at 134 x g for 3 min and then incubated at 37°C for 24 h. The non-adherent THP-1 cells were removed by gently washing the wells three times with RPMI medium. The adherent THP-1 cells were microscopically quantified (magnification, ×100) in four randomly selected visual fields of each well. Images were captured using an Axiovert 200 inverted fluorescence microscope (Carl Zeiss, Jena, Germany).

### Western blotting

Western blotting was performed, as previously described (19). Briefly, the HSFs and NFs (3×10^5^ cells/well) were treated with either emodin (40 *μ*g/ml) or DMEM and incubated at 37°C in a humidified atmosphere containing 5% CO_2_ for 24 h. The cells were lysed using radioimmunoprecipitation assay lysis buffer (Beyotime Institute of Biotechnology, Haimen, China) supplemented with 1 mM phenylmethylsulfonyl fluoride (Adamas, Shanghai, China). The protein samples were subsequently separated by 12% sodium dodecyl sulfate (SDS)-polyacrylamide gel electrophoresis and transferred to Hybond enhanced chemiluminescence (ECL) membranes (GE Healthcare Life Sciences, Chalfont, UK). The membranes were blocked in 6% nonfat milk dissolved in TBST buffer containing 10 mM Tris-HCl, (pH 8.0), 150 mM NaCl and 0.05% Tween 20 and incubated with primary antibodies, including rabbit anti-mouse polyclonal TNF-α antibody (1:1,000; #3707), rabbit anti-mouse monoclonal IL-6 antibody (1:1,000; #12912), rabbit anti-mouse polyclonal MCP-1 antibody (1:1,000; #2029), rabbit anti-mouse polyclonal PI3K antibody (1:1,000; #4257), rabbit anti-mouse polyclonal p-PI3K antibody (1:1,000; #4228), rabbit anti-mouse polyclonal Akt antibody (1:1,000; #9272) and rabbit anti-mouse polyclonal p-Akt antibody (1:1,000; #4060) (Cell Signaling Technology, Inc., Danvers, MA, USA) at 4°C overnight. The membranes were then incubated with goat anti-rabbit immunoglobulin G secondary antibody coupled with horseradish peroxidase at room temperature for 2 h. The blots were developed using an ECL system (GE Healthcare Life Sciences). For re-probing, the blots were incubated in stripping buffer containing 100 mM 2-mercaptoethanol, 2% SDS and 62.5 mM Tris-HCl (pH 6.7) at 50°C for 30 min.

### Statistical analysis

SPSS version 13.0 (SPSS Inc., Chicago, IL, USA) was used for all statistical analyses. Significant differences were calculated from the means of triplicate samples using a factorial design analysis of variance. P<0.05 was considered to indicate a statistically significant difference.

## Results

### Histopathological assessment

**Figure 1:**
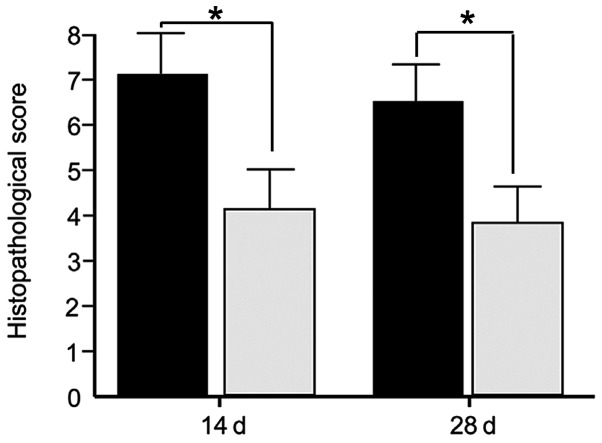
Histopathological scores of hypertrophic scar tissue following treatment with emodin. ^*^P<0.05. The data are presented as the mean ± standard error of the mean. Black, experimental group; grey, control group.

HS is marked by the accumulation of a substantial quantity of abnormally secreted ECM. HS tissue is characterized by a deeper collagen fiber thickness, more disorganized collagen structure and more severe inflammatory response compared with normal scar tissue (1). The present study measured the SEI, collagen structure and inflammation and scored them between 0 to 3, as described previously. The three parameters were lower, by at least one grade, in the HS specimens treated with emodin compared with the control tissue. Additionally, the collagen whorls disappeared and the level of inflammatory cells was markedly reduced. Treatment with emodin significantly altered the histological status and histopathological scores, compared with the control group on day 14 post-incision (Fig. 1). Between days 14 and 28 post-incision, treatment with emodin was terminated, however, the histopathological scores only marginally improved.

### Reduced adhesion of inflammatory cells in HS

**Figure 2:**
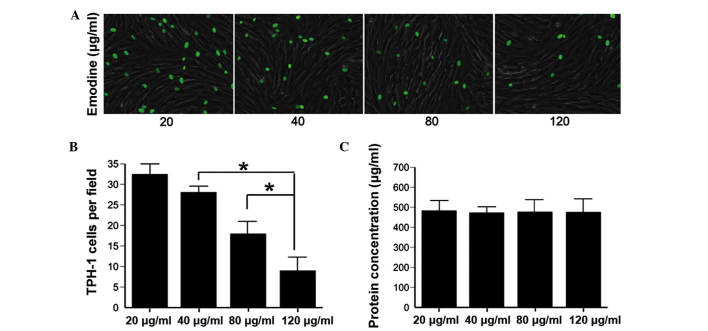
Attenuation of adhesion of the THP-1 human acute monocytic leukemia cell line and HSFs by emodin. (A) Representative images of cell-cell adhesion assays. Green spots represent calcein AM-labeled adherent THP-1 cells. (B) Quantification of adherent THP-1 cells. The data are presented as the mean ± standard error of the mean. ^*^P<0.05. (C) Total protein concentration of HSFs following treatment with 20, 40, 80 and 120 *μ*g/ml emodin for 24 h. The data are presented as the mean ± standard error of the mean. HSF, hypertrophic scar fibroblast.

During the early proliferative phase, the recruitment, adhesion, retention and interaction of inflammatory cells with topical fibroblasts results in an excessive and chronic inflammatory response, which contributes to the formation of HS (4,7). To investigate the direct interaction between inflammatory cells and fibroblasts treated with various concentrations of emodin, a cell-cell adhesion assay was performed using THP-1 cells, a typical type of monocyte. Emodin effectively reduced the adhesion and mutual reaction of THP-1 and HSFs, in a dose-dependent manner (Fig. 2A and B). These results confirmed, to a certain extent, that emodin inhibited the mechanical stress-induced HS inflammatory response by reducing the cell-cell interaction between inflammatory cells and HSFs.

### Cytotoxicity of emodin

**Figure 3:**
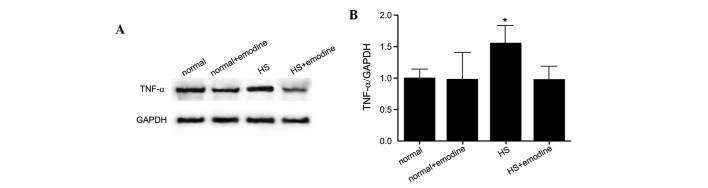
Protein expression levels TNF-α in each group determined by (A) western blot analysis and presented as a (B) graph. ^*^P<0.05, vs. normal, normal + emodine and HS + emodine. The data are presented as the mean ± standard error of the mean. HS, hypertrophic scarring; TNF, tumor necrosis factor.

**Figure 4:**
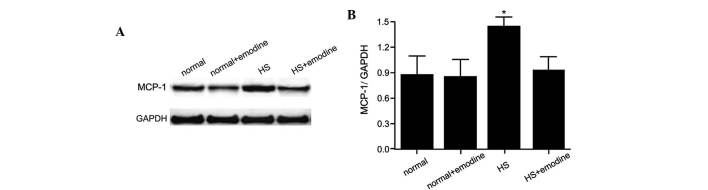
Protein expression levels of MCP-1 in each group determined by (A) western blot analysis and presented in a (B) graph. ^*^P<0.05, vs. normal, normal + emodine and HS + emodine. The data are presented as the mean ± standard error of the mean. HS, hypertrophic scarring; MCP, monocyte chemoattractant protein.

**Figure 5:**
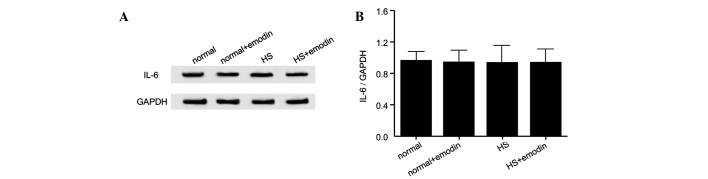
Protein expression levels of IL-6 in each group determined by (A) western blot analysis and presented as a (B) graph. The data are presented as the mean ± standard error of the mean. HS, hypertrophic scarring; IL, interleukin.

**Figure 6:**
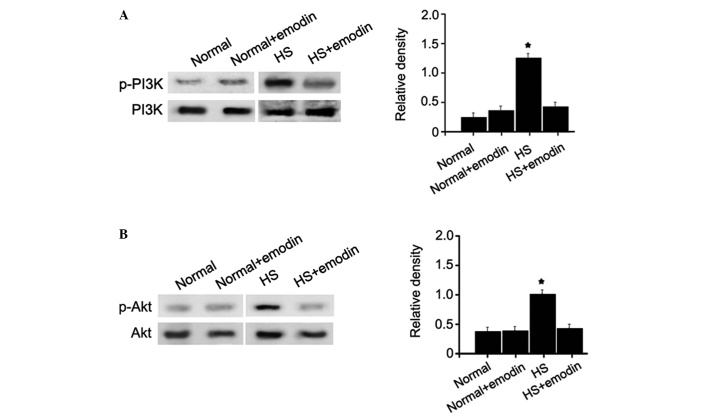
Reduced levels of p-PI3K and Akt in response to treatment with emodin. (A) Expression levels of p-PI3K/PI3K ^*^P<0.01. The data are presented as the mean. (B) Expression levels of p-Akt/Akt. ^*^P<0.01, vs. normal, normal + emodine and HS + emodine. The data are presented as the mean ± standard error of the mean. HS, hypertrophic scarring; p-, phosphorylated; PI3K, phosphoinositide 3-kinase.

Emodin has been widely used clinically with few side effects. To investigate the cytotoxicity of emodin *in vitro*, a total protein assay was performed on NFs treated with emodin. No significant change was observed in the quantity of total protein as the concentration of emodin increased (Fig. 2C; P>0.10) and no significant changes were observed in the expression levels of TNF-α, IL-6, MCP-1, phosphorylated (p-) PI3K/PI3K and p-Akt/Akt in the control NFs (Figs. 3–6). These results indicated that emodin was safe for the treatment of HS.

### Attenuation of the expression of HS inflammatory cytokines by emodin

The levels of inflammatory cytokines, which are closely associated with the HS inflammatory response, were also assessed in the present study. The protein expression levels of TNF-α, MCP-1 and IL-6 in NFs, NFs treated with emodin and HSFs and HSFs treated with emodin are presented in Figs. 3–5, respectively. The cytokine expression levels were significantly increased in the HSF group compared with the NF group. However, in response to treatment with emodin, the expression levels of these cytokines were not markedly altered compared with the controls. These findings demonstrated that emodin effectively inhibited the HS inflammatory response by attenuating the production of inflammatory cytokines.

### Reduced activation of the expression of PI3K and Akt

Numerous studies have demonstrated that the PI3K/Akt signaling pathway is a crucial mediator in the development of fibrotic diseases, which often involve inflammatory responses (20–24). To determine the underlying mechanism by which emodin inhibits the mechanical stress-induced inflammation of HS, the relative phosphorylation levels of PI3K and Akt proteins were examined in HSFs and NFs, with or without emodin treatment. The levels of p-PI3K and p-Akt were significantly higher in the HSFs compared with the control group (Fig. 6). Treatment with emodin had no significant effects on the activation of PI3K and Akt in NFs. However, in the HSFs, treatment with emodin markedly reduced the activation of these two proteins to levels close to normal. These findings support the hypothesis that emodin has inhibitory effects on the mechanical stress-induced inflammation of HS by downregulating the PI3K/Akt signaling pathway.

## Discussion

HS is a fibroproliferative disorder, which can lead to unsightly scarring or deformities of the organs and extremities. There is currently no effective treatment for HS, partly due to limited understanding of the mechanisms underlying HS. Therefore, the identification of novel antifibrotic activation drugs is essential.

A previous study demonstrated that inflammation and mechanical stress are crucial in the initiation, maintenance and progression of HS (17). Mechanical stress-induced HS in mice is a novel and well-characterized model, which is used in the investigation of HS and was used in the present study (16). Histopathological examinations indicated that collagen fibers were thicker and larger with a more disorganized arrangement and collagen whorls developed in mechanical stress-induced HS. In addition, varying degrees of inflammatory responses developed, which was marked by the recruitment and adhesion of monocytes and mast cells. Certain anti-inflammatory drugs, including corticosteroids, can effectively inactivate fibroblasts and reduce the inflammatory response in HS (25). However, due to severe adverse effects and high costs, the use of these drugs is limited for the long term and widespread treatment of HS.

The present study examined the effects of emodin, a cheaper drug with fewer side effects, in HS treatment. Emodin is extracted from the Chinese herb rhubarb and has been confirmed as an effective drug for the treatment of fibrosis and the suppression of inflammatory responses (14,26). In response to treatment with emodin, the histopathological score of the HS was markedly reduced. Furthermore, *in vitro* cell adhesion assays of the THP-1 and HSFs further revealed that emodin attenuated the retention of monocytes and reduced the contact and interaction between the cells in a dose-dependent manner. Therefore, it was hypothesized that HS inflammation can be inhibited by emodin through the suppression of inflammatory cell recruitment, adhesion, retention and activation.

Several types of cytokine are associated with the HS inflammatory response, including TNF-α, IL-6, transforming growth factor-β and MCP-1. In order to investigate the effects of emodin on inflammatory cytokines in HS, the protein expression levels of TNF-α, IL-6 and MCP-1 were determined by western blotting. Previous studies have demonstrated that the expression levels of TNF-α or the TNF-α receptor are markedly upregulated in HS (9,27,28) and matrix metalloproteinase (MMP)-1 and MMP-3 are downregulated, which attenuates the excessive accumulation of collagen formed in hypertrophic scars by suppressing MMPs under the control of IL-6 (29). These findings are consistent with those of the present study, which demonstrated that the expression levels of TNF-α and MCP-1 were markedly increased, but the expression of IL-6 was not significantly altered in HS. In response to treatment with emodin, the expression levels of TNF-α and MCP-1 were gradually restored close to normal levels and the expression of IL-6 remained unchanged. These results indicated that, to a certain extent, HS inflammation can be inhibited by emodin by suppressing the production of inflammatory cytokines.

The PI3K/Akt signaling pathway has a key role in the inflammatory response and, to a certain extent, the levels of p-PI3K and p-Akt determine the strength of the inflammatory response and the formation of HS (30). To further investigate the mechanisms by which emodin inhibits HS inflammation in the present study, the levels of p-PI3K and p-Akt were determined. The phosphorylation of PI3K and Akt in HS was markedly reduced following treatment with emodin, which was in accordance with the results of previous studies investigating the effects of emodin on other systems (31,32). These results suggested that the PI3K/Akt signaling pathway may mediate the inhibitory effects of emodin on HS inflammation.

In conclusion, the present study demonstrated that emodin may inhibit mechanical stress-induced HS inflammation by reducing histopathological scores, attenuating inflammatory cell recruitment and adhesion and suppressing the secretion of inflammatory cytokines by inhibiting the PI3K/Akt signaling pathway. However, whether emodin can be used clinically to treat HS, and whether it acts upon other signaling pathways to affect HS remains to be elucidated. Therefore, further detailed studies are required to evaluate the therapeutic use of emodin.
